# Inhibition of Tumor Growth and Modulation of Antioxidant Activity of Rhodoxanthin Isolated from *Taxus baccata* Aril against B16F10 Murine Malignant Melanoma

**DOI:** 10.3390/antiox11112264

**Published:** 2022-11-16

**Authors:** Daria-Antonia Dumitraş, Alexandra Iulia Dreanca, Emoke Pall, Adrian Florin Gal, Vasile Rus, Andreea Georgiana Morohoschi, Mihaela Cotul, Monica Irina Nan, Sanda Andrei

**Affiliations:** 1 Department of Preclinical Sciences, Faculty of Veterinary Medicine, University of Agricultural Sciences and Veterinary Medicine, 400374 Cluj-Napoca, Romania; 2 Department of Clinical Sciences, Faculty of Veterinary Medicine, University of Agricultural Sciences and Veterinary Medicine, 400374 Cluj-Napoca, Romania

**Keywords:** B16F10 murine melanoma, *Taxus baccata*, rhodoxanthin, tumor growth inhibition, antioxidant activity

## Abstract

Malignant melanoma is the most aggressive type of skin cancer, and due to the numerous limitations of current treatment methods, there is an urgent need to develop novel approaches for both the prevention and treatment of malignant melanoma, with research-oriented bioactive substances representing a notable first step. The current study decided to expand on previous rhodoxanthin research by investigating the possible anti-tumor effect as well as the effect on the antioxidant status in the case of murine melanoma in an experimental model. The 21-day study was carried out on female C57BL/6J mice. On the first day of the experiment, they were subcutaneously inoculated with 10^6^ B16F10 cells and were given rhodoxanthin orally until the end of the study. Rhodoxanthin supplementation significantly reduced tumor growth (42.18%) and weight (15.74%). Furthermore, the epidermal growth factor (EGF) activity was reduced and the concentration of 8-OHdG dropped in the treated melanoma-bearing mice compared to the untreated ones, demonstrating the role of rhodoxanthin in slowing tumor growth, one of the mechanisms being the reduction of EGF level and the decrease of DNA oxidation. The administration of rhodoxanthin determined variations in antioxidant enzymes, both at the plasma level and at the tissue level.

## 1. Introduction

Malignant melanoma is the most aggressive type of skin cancer, with the worst prognosis, and it affects a younger population than most cancers [1]. René Laënnac first characterized cutaneous malignant melanoma in 1806, proving an etiology that originates from the alteration and uncontrolled development of melanocytes which resists the most conventional remedies, resulting in a growing public health problem [2,3]. Being the 19th most frequent skin neoplasia, every year, around 200,000 new cases of primary malignant melanoma cancer are reported worldwide (excluding non-melanoma skin cancers), with around 55,500 deaths reported each year. According to the Skin Cancer Foundation, the number of new invasive melanoma diagnoses grew by 44 percent over the last decade (2011–2021). Skin cancers are also among the most commonly diagnosed diseases in animals. The canine species has been identified as the species with the highest prevalence of skin malignancies, accounting for one-third of all tumors diagnosed in it [4]. In the case of cats, skin cancers play a crucial role, accounting for one-quarter of all neoplasias diagnosed in this species. Annual estimations of the incidence of cutaneous neoplasia in dogs and cats have been recorded, with the dog having 450 cases out of 100,000 and the cat having 120 cases out of 100,000 [5,6,7,8]. Older dogs appear to be the most typically affected, with an average age of 9 years documented in the literature.

Unfortunately, the current treatment methods such as cryotherapy, external surgery, radiation therapy, chemotherapy, photodynamic therapy, biological therapy, and targeted medication therapy such as vemurafenib, dabrafenib, and trametinib, have multiple limitations due to the several side effects they can cause, multidrug resistance and last but not least, the high cost. In view of these aspects, the need to discover new methods for both prevention and therapy of malignant melanoma, with research-oriented bioactive substances representing a noteworthy step due to the multiple beneficial effects that plant-derived chemicals can exert is urgent. The fact that plant-derived chemicals have active molecules that alter cell proliferation, apoptosis, metastasis, and angiogenesis, and that they may be employed against cancer safely, efficiently, and with low side effects, is a huge advantage [9,10,11,12]. Furthermore, their ability to defend against some of the negative effects of radiotherapy and chemotherapy should not be overlooked.

Also known as European or English yew, *Taxus baccata*, and its numerous related cultivars, e.g., ‘Elegantissima’ and ‘Washingtonii’ is an evergreen coniferous native to Europe [13]. It became popular mostly because of its poisonous active ingredients (taxine alkaloids) occurring in varying amounts throughout the plant’s sections, with several incidents of poisoning reported in recent years [14,15,16]. However, scientists also wanted to find bioactive substances with beneficial effects contained in this plant, and as a result, they discovered Taxol (with the generic name Paclitaxel), which was isolated for the first time from the bark of *Taxus brevifolia Peattie* (Pacific yew), to be one of the most valuable bioactive substances due to its antitumoral characteristics, particularly in relation to ovarian and breast cancer. More recently, new developments in the composition of arils have aroused the curiosity of scientists in the field. Because of their high concentration of vital micro- and macro-elements, high-quality proteins, and low amount of simple carbs, arils could represent a potentially valuable component of the food diet [17]. Carotenoids, particularly those with a retro structure, such as rhodoxanthin, but also, in small amountlycopene and zeaxanthin, have been identified as one of the most essential components of these fruits. Rhodoxanthin is a rare pigment found in nature, formerly detected in the feathers of some fish and birds [18,19]. An increasing amount of research demonstrates the critical importance of carotenoids in numerous multifactorial processes in both animal and human organisms, as well as their capacity to interact with radical species, thereby reducing the negative effects these species have on the emergence and development of diseases like degenerative diseases, age-related macular degeneration, cardiovascular diseases, and a significant number of neoplasms.

Following a previous study [20] that demonstrated an important antiproliferative and proapoptotic effect of rhodoxanthin on B16F10 murine melanoma cells in vitro, it was decided to extend the research by looking into the possible anti-tumor effect of rhodoxanthin in the case of murine melanoma in an experimental model. Given these aspects and taking into consideration the lack of information regarding the potential antitumoral activity of rhodoxanthin, we aimed our focus on studying this retro-carotenoid isolated from the flesh of the red aril of *Taxus baccata* emphasizing its activity against B16F10 murine malignant melanoma.

## 2. Materials and Methods

### 2.1. Chemicals and Reagents

The kits used during the present study were obtained from Elabscience Biotechnology Inc., Texas, HT, USA. The remaining reagents used were acquired from Sigma Aldrich (Darmstadt, Germany) and Merck (Darmstadt, Germany).

### 2.2. Identification and Collection of Plant Materials

Red berries (arils) of *Taxus baccata* L. were collected from plants growing in natural habitats, in the autumn (September–October) of 2020 from the University of Agricultural Sciences and Veterinary Medicine campus, Cluj-Napoca (46°46′0.01″ N, 23°36′0.00″ E), Cluj County, Romania. After the fruits were collected, the toxic part (seed) was removed manually and the whole quantity of fruits was divided into sample bags, each weighing approximately 20 g FWA (fresh weight arils), and stored in the freezer (−80 °C) until further processing.

### 2.3. Plant Extract Preparation

Pigment extraction was performed using a mixture of methanol: ethyl acetate: petroleum ether (1:1:1, *v*/*v*/*v*), and the total carotenoid content was determined, the separation and the identification of the carotenoids were carried out by open-column chromatography (OCC) in an open column of silica gel and eluted with n-hexane–acetone–methanol 85:15:1 (*v*/*v*/*v*). Three colored bands were partitioned, following being separately collected and concentrated in a rotary evaporator (≤35 °C) and dissolved in a known volume of hexane. In order to identify the pigments, present in the separated fractions, the absorption spectra in the range of 300–600 nm were recorded. Pigment identification was based on comparing the characteristic absorption spectra with previous literature data [21,22,23]. Also, HPLC carotenoid separation was accomplished on the total extract and the fractions previously separated through OCC, with all chromatograms monitored at 450 nm.

After identifying the major carotenoid, rhodoxanthin, the extraction was repeated until the required amount of rhodoxanthin was obtained. The ether phase was evaporated, and the resulting oleoresin was suspended in MCT (medium-chain triglyceride) oil at the indicated dosage: 7 mg rhodoxanthin/kg b.w. Each mouse treated with rhodoxanthin received the required dosage according to its weight through oral administration after being dissolved in 100 µL of MCT oil.

### 2.4. Cell Line and Culture

The Institute of Oncology “Prof.Dr. Ion Chiricuță” Cluj-Napoca, Romania provided the B16F10 murine melanoma cells. The cells were incubated for five days in Dulbecco’s Modified Eagle Medium supplemented with 10% fetal bovine serum and 100 IU/mL penicillin in a humidified incubator containing 5% CO_2_ (Advantage-Lab, Schilde, Belgium) at 37 °C in a humidified atmosphere. Previous to this experiment, rhodoxanthin was tested in vitro on the same cell line [3]. Prior to the inoculation, cells were harvested by trypsinization and washed three times with PBS.

### 2.5. Experimental Animals

Experiments were performed using 40 female C57BL/6J mice of 8–10 weeks, weighing approximately 20 g, that were obtained from the Institute of Oncology “Prof.Dr. Ion Chiricuță” Cluj-Napoca, Romania. The animals were randomly divided into 5 equal groups. All animals were housed according to ISO 10993-2 standards [24] and Directive 63/2010/EU in individually ventilated cages, at 25 ± 2 °C and 55% ± 10% relative humidity, under a 12 h light-dark cycle, in the authorized Laboratory Animal Establishment (University of Agricultural Sciences and Veterinary Medicine Cluj-Napoca). Also, the mice were provided ad libitum access to standard rodent food (purchased from Cantacuzino Institute, Bucharest, Romania) and filtered water. All animal procedures were approved by Regional Sanitary Veterinary and Food Safety Authority (no. 276/12.11.2021) and followed the guidelines of Directive 63/2010 and national law no. 43/2014. Surgical procedures were performed according to ISO 10993-6. The mice were acclimatized for one week before the experimental procedures.

### 2.6. Tumor Model and In Vivo Treatment Regimen

A total of 40 adult female C57BL/6J mice aged 8–10 weeks were divided into 5 groups as follows. Tumor-bearing mice were injected into the previously shaved flanks, subcutaneously, with 1 × 10^6^ B16F10 melanoma cells/mouse.

Group 1 (*n* = 8)–Control: received Phosphate buffer saline (PBS) 0.9%, subcutaneously;

Group 2 (*n* = 8)—MCT oil: received 100 µL of MCT oil, orally administered, for 21 days;

Group 3 (*n* = 8)—Rhodoxanthin: received 100 µL of MCT oil/day, orally, for 21 days, containing the according dosage of rhodoxanthin for each mouse (7 mg rhodoxanthin/kg b.w.)

Group 4 (*n* = 8)—Melanoma: inoculated with 1 × 10^6^ B16F10 cells/100 µL PBS/mouse.

Group 5 (*n* = 8)—Melanoma + Rhodoxanthin: inoculated with 1 × 10^6^ B16F10 cells/100 µL PBS/mouse and received 100 µL of MCT oil/day, orally, for 21 days containing the according dosage of rhodoxanthin for each mouse (7 mg rhodoxanthin/kg b.w.).

On the first day of the experiment, each mouse was weighed and marked. The mice belonging to the Melanoma group and Melanoma + Rhodoxanthin group were inoculated with 1 × 10^6^ cells/100 µL PBS/mouse, in the previously shaved flanks. The Control group received 0.9% PBS subcutaneously and the MCT oil group received via gavage 100 µL of MCT oil. The Rhodoxanthin group and Melanoma + Rhodoxanthin group were given, via gavage, the required dosage of rhodoxanthin (7 mg/kg b.w.) in 100 µL of MCT oil. The administration of MCT oil and Rhodoxanthin was continued for each day of the following 21 days of the experiment.

During the experiment, body weight mass, survival rate, infection prevention, and tumor size were closely monitored. The size of the tumors was measured using Vernier calipers. The width (a) and length (b) of the tumors were measured for the determination of tumor volume according to the formula V = ba^2^/2 [25]. The animals were humanely euthanized under general anesthesia by cervical dislocation at the end of the experiment (day 22), in accordance with international norms [26]. Prior to euthanasia, the animals were weighed and blood samples from the orbital sinus using EDTA (Ethylenediamine Tetra-Acetic Acid) 10% as an anticoagulant, were obtained for future analysis. Roughly, 0.5–1 mL of blood was obtained from each animal and kept in previously specified 2 mL microtubes for hematological and biochemical analyses. Samples of liver, skin, and tumors were collected from all the animals in order to perform biochemical determinations. Tumors from the Melanoma and Melanoma + Rhodoxanthin groups were excised, weighed, and preserved in paraformaldehyde for histopathological examination. The tumor inhibition ratio was calculated using the formula IR (%) = [(A − B)/A] × 100, where A represents the average tumor weight of the melanoma group and B represents the average tumor weight of the melanoma + rhodoxanthin group [27].

### 2.7. Hematological and Biochemical Analysis

The hematological analyses were performed on whole blood using an automatic veterinary hematological analyzer (Abacus Junior Vet 5 Diff, DIATRON MI ZRT., Hungary) to assess the complete blood count, namely: red blood cells (RBC), hemoglobin, hematocrit, mean corpuscular volume (MCV), mean corpuscular hemoglobin concentration (MCHC), total and differential leukocyte and platelet counts. Further, for the investigation of the biochemical analysis and the activity of numerous oxidative stress-related enzymes plasma was obtained by blood centrifugation at 4000 RPM for 10 min.

Parameters such as catalase (CAT) (Catalase CAT Activity Assay Kit), superoxide dismutase (SOD) (Superoxide Dismutase SOD Assay Kit), glutathione peroxidase (GSH-Px) Glutathione Peroxidase (GSH-Px Activity Assay Kit), total antioxidant status (TAS) (Total Antioxidant Capacity (T-AOC) Colorimetric Assay Kit), were determined from the plasma samples using specifical kits (Elabscience Biotechnology Inc., Texas, HT, USA).

An additional biochemical investigation was performed on tissue samples previously collected from the mice (skin, liver, tumor). Protein quantification was the first step in facilitating the investigation of other tissue parameters. This was performed according to the biuret method [28], and the results were expressed as mg-protein/mL homogenate. The same enzymes that were determined in the case of plasma, were also determined from protein extracts, using specific kits. Enzyme analysis results were given in U/mg-protein. All the biochemical analyses were validated and performed via spectrophotometry using the microplate spectrophotometer SPECTROstar^®^ Nano (BMG Labtech, Germany).

### 2.8. ELISA Technique for Determining Carcinogenesis Indicators and DNA Oxidation Level

By means of the ELISA immunochemical technique, we determined two important factors in tumor development, namely: epidermal growth factor (EGF) which plays an important role in cell growth, cell differentiation, proliferation, and migration, especially in the case of epithelial cells, by binding to its receptor, EGFR (Epidermal Growth Factor Receptor). Mutations leading to EGFR/EGF overexpression have been linked to several types of cancer, both in animals and humans, and are closely correlated with tumor growth, differentiation, and metastasis, via a variety of mechanisms including increased tyrosine kinase activity, altered autophagy activity, or by stimulating the expression of matrix metalloproteinases (MMP-MMP1, MMP9) that facilitate cell progression and invasion [29].

The second parameter targeted was 8-hydroxy-2′-deoxyguanosine (8-OHdG). Numerous studies have provided substantial data by closely monitoring oxidative degradation, finding that it occurs permanently on cell membrane lipids, proteins, and DNA. In nuclear and mitochondrial DNA, 8-hydroxy-2′-deoxyguanosine (8-OHdG) or 8-oxo-7,8-dihydro-2′-deoxyguanosine (8-oxodG) is one of the predominant forms of alterations induced by free radicals, thus being widely used as a biomarker for oxidative stress and carcinogenesis [30]. 8-OHdG production can result in G:C-T:A transversions, which are linked to tumor development and progression, cellular aging, and a variety of degenerative illnesses [31]. Both determinations were carried out with the use of particular kits (Elabscience Biotechnology Inc., Texas, HT, USA).

### 2.9. Histopathology Analysis

The tumor samples collected were fixed in 10% buffered formalin for three days before going through a stepwise dehydration method (ethylic alcohol at 70%, 96%, and 100%) for one hour at a time. The clarification was performed in three 1-h-long 1-butanol baths before being embedded in paraffin. For histological analysis, 5 µm tissue sections were cut using a Leica rotary microtome (RM2125, Wetzlar, Germany) and stained with Goldner’s trichrome [32]. The slide’s evaluation was accomplished using an Olympus BX-41 microscope (Olympus, Tokyo, Japan) attached to the Olympus E330 camera (Olympus, Japan). Tumor samples were assessed for size, symmetry, delimitations, development, necrosis/ulceration, inflammatory infiltration, regression, cellular atypia, mitoses, melanization, and isolated cell proliferation.

### 2.10. Statistical Analysis

GraphPad Prism 8 statistics program (San Diego, CA, USA) was used for the statistical calculations. Data statistical analyses were achieved by using one-way ANOVA. Differences between the experimental groups and the controls were evaluated with a level of significance set at *p* < 0.05. All the determinations were performed in triplicate, and the results were expressed as arithmetical mean values ± standard deviations.

## 3. Results and Discussions

### 3.1. Rhodoxanthin’s Effect on B16F10 Murine Melanoma Growth in C57BL/6J Mice

**Figure 1:**
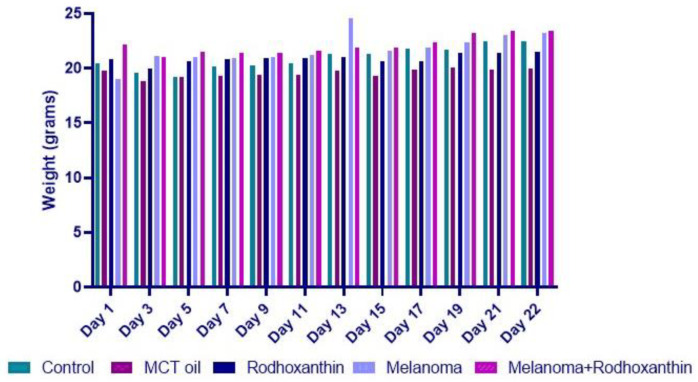
The weight variation of individuals included in the study (Control: received (PBS) 0.9%, sc.; MCT oil: 100 µL of MCT oil, orally administered, for 21 days; Rhodoxanthin: received 100 µL of MCT oil/day, orally, for 21 days; Melanoma: inoculated with 1 × 10^6^ B16F10 cells/100 µL PBS/mouse; Melanoma + Rhodoxanthin: inoculated with 1 × 10^6^ B16F10 cells/100 µL PBS/mouse and received 100 µL of MCT oil/day, orally, for 21 days containing the according dosage of rhodoxanthin for each mouse).

During the experiment, no changes in the individual’s general condition were observed; however, in general, in experimental models aimed at inducing various types of neoplasia, cachexia is a frequently encountered syndrome, representing progressive weakening through muscle mass loss and accounting for 20–30% of deaths in both experimental, animal, and human models [33]. Throughout the study, 11 weightings were performed, as can be seen in Figure 1.

**Figure 2:**
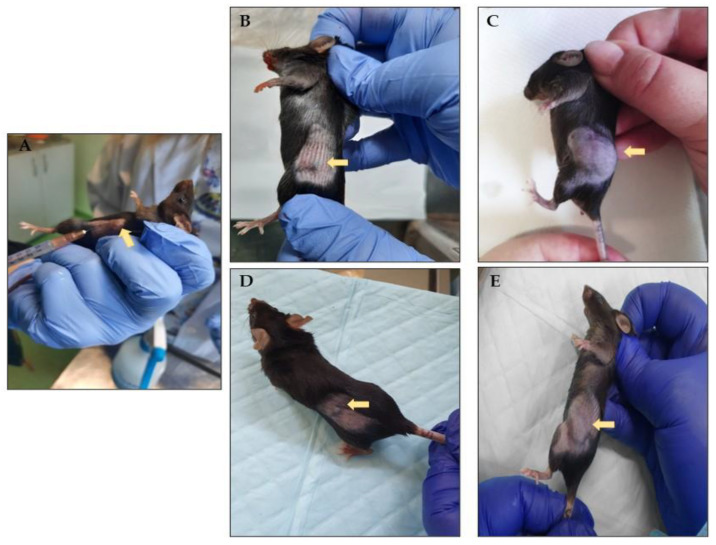
Tumor growth variation following the B16F10 cells inoculation (**A**). First measurable tumors in melanoma group (**B**) and melanoma + rhodoxanthin group (**D**). Last measurement of the tumors in melanoma group (**C**) and melanoma + rhodoxanthin group (**E**).

Other symptoms such as decreased mobility, lethargy, piloerection, or partial eye closure, or adverse effects such as diarrhea, common in similar experiments, were not seen in any of the individuals, indicating that rhodoxanthin therapy did not cause undesirable effects or toxicity. Mice were examined daily to monitor and determine the timing of the appearance of tumors in the area where the murine melanoma cells were injected on the first day of the experiment. On the eighth day, the first signs of tumor growth were noticed in a number of individuals. Tumors were measured (length and width) at two-day intervals until the end of the experiment. Throughout the study, we discovered that despite the identical number of inoculated cells, growth varied between individuals within the same group (Figure 2).

At the end of the study, on day 22, biological blood samples were collected from each individual for biochemical and hematological determinations. Following euthanasia, tumors were also measured and collected for biochemical, hematological, and histopathological investigations.

**Figure 3:**
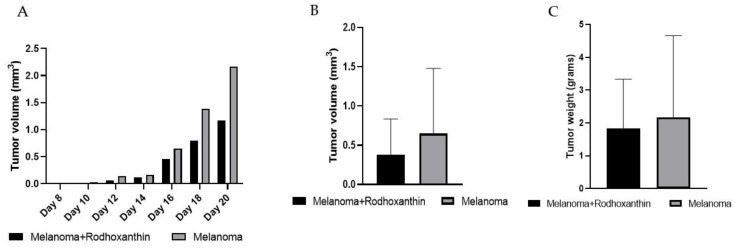
Effect of rhodoxanthin on solid B16F10 melanoma tumor growth in C57BL/6J mice. (**A**) Tumor volume variation throughout the study. (**B**) Significant inhibition of tumor volume between the untreated group (0.648 ± 0.829) and the treated group (0.376 ± 0.457). Each value represents the mean ± SD, *n* = 8). (**C**) Tumor weight inhibition at a value of 15.48% recorded after the administration of 7 mg/kg/day rhodoxanthin.

Following the measured values during the investigation and the determination of the tumor volume, we acquired the data displayed in Figure 3. As can be observed, the average tumor volume increased exponentially during the experiment. In terms of tumor volume variation, there was no statistically significant difference (*p* > 0.005) between the two main groups. However, the difference between the average tumor volumes corresponding to the untreated group (0.648 ± 0.829), respectively the average tumor volumes corresponding to the group treated with rhodoxanthin (0.376 ± 0.457), cannot be neglected, there being a difference of 41.97%, in favor of the melanoma group.

Also, tumor growth differed within the same group, a feature that is most likely due to individual characteristics. Thus, the weight of the tumors in the melanoma group ranged from 0.55 g to 6.72 g, whereas the weight of the tumors in the melanoma + rhodoxanthin group ranged from 0.53 to 3.98 g. However, the difference in tumor weight between groups was not statistically significant (*p* > 0.05). Similar to the tumor volume, in the case of the average tumor weights collected after euthanasia, we can observe a difference between the treated (1.828 ± 1.506) and the untreated group (2.163 ± 2.497) so that the average tumor mass after excision, in the case of the treated group, was 15.74% lower than in the case of the untreated group.

### 3.2. Hematological Analysis

**Figure 4:**
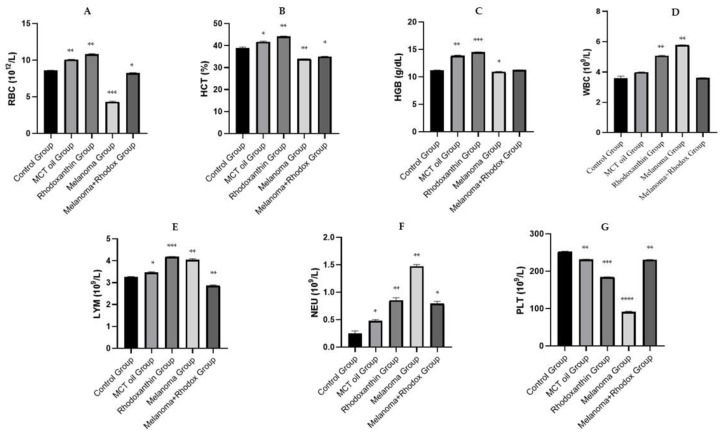
Hematological profile data. (**A**) Red blood cell count. (**B**) Hematocrit. (**C**) Hemoglobin. (**D**) White blood cell count. (**E**) Total lymphocyte count. (**F**) Total neutrophils count. (**G**) Platelet count. Values are expressed as mean ± standard deviation SD from three independent experiments. Statistical significance level * *p* < 0.05, ** *p* < 0.01, *** *p* < 0.01 and **** *p* < 0.0001.

The complete blood count can reveal a multitude of imbalances and general pathological conditions in the body by changing leukocyte, erythrocyte, and/or platelet parameters. As a result, the hematological examination can be intimately correlated to the overall health of the monitored individual. The hematological table is considered to be the primary indicator of cancer development, and it is an important aspect in determining a favorable or negative prognosis [34]. The hematological profile evaluation data are shown in Table 1 and Figure 4.

Regarding the total number of platelets, drastic thrombocytopenia was registered in the case of the melanoma group, with a statistically significant value compared to the control group (91.333 ± 1.52 vs. 252.12 ± 1.42; *p* < 0.0001). These changes are consistent with data published in other studies specifying severe platelet depletion in individuals with cutaneous melanocytic tumors [35,36]. The value of platelets in the melanoma + rhodoxanthin group was close to that of the control (230.643 ± 0.66), as was the value of the rhodoxanthin group (184.5 ± 0.51).

The overall number of white blood cells remains relatively constant, with a slight elevation in the melanoma group compared to the control group (5.777 ± 0.12 vs. 3.603 ± 0.14, *p* < 0.01). This increase is primarily the result of an increase in the number of neutrophils. The melanoma + rhodoxanthin group’s leukocyte value is comparable to the control group (3.626 ± 0.05, *p* < 0.01). Similarly, to prior studies (Boussabbeh et al., 2016; Kamran et al., 2018), untreated tumor-bearing mice were anemic during the experiment, with the total number of red blood cells being almost two times lower than in the control group (4.332 ± 0.05 vs. 8.593 ± 0.03). Furthermore, hemoglobin and hematocrit levels of individuals from the melanoma group were also decreased by 2.06% for hemoglobin (10.943 ± 0.05 vs. 11.210 ± 0.04), respectively 11.77% for hematocrit (33.916 ± 0.13 vs. 38.926 ± 0.41) compared to the values of the control group. Rodhoxantin administration significantly increased the values of red blood cells, hemoglobin, and hematocrit in treated tumor-bearing mice.

### 3.3. The Determination of Antioxidant Enzyme Activity

The biochemical profile consisted of the determination of some parameters both from plasma (CAT, SOD, TAS, GPx) and from tissues (Total proteins, CAT, SOD, TAS, GPx). Although tumor cells are producers of large amounts of reactive oxygen species, according to some studies [37,38,39], the value of antioxidant enzymes in tumor cells is generally lower due to their reduced ability to defend themselves against free radicals, resulting in an oxidant-antioxidant imbalance, namely the state of oxidative stress.

**Figure 5:**
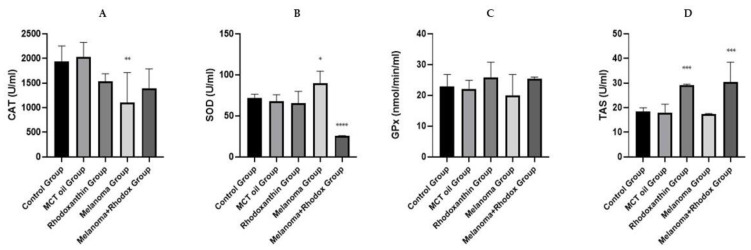
Enzymatic antioxidant activity and total antioxidant status in plasma. (**A**) Catalase activity. (**B**) Superoxide-dismutase activity. (**C**) Glutathione-peroxidase activity. (**D**) Total antioxidant status. Values are expressed as mean ± standard deviation SD from three independent experiments. Statistical significance level * *p* < 0.05, ** *p* < 0.01, *** *p* < 0.01 and **** *p* < 0.0001.

Figure 5 shows the enzyme activity values determined from the plasma of the individuals under study. Thus, regarding catalase determined from plasma, both in the case of individuals from the melanoma group and those from the melanoma + rhodoxanthin group, the average value of this enzyme was low, compared to the average value corresponding to the control group (139.3 ± 608.66 vs. 194.02 ± 313.29, 110.2 ± 308.46 vs. 194.02 ± 313.29). These data are consistent with data published by other researchers reporting catalase as an enzyme with reduced activity in tumor-bearing individuals [40]. Superoxide-dismutase, an enzyme that has been associated in the specialized literature with a varied activity regarding neoplasms, initially mentioned a low level of its activity in tumor cells, but later research mentioned an increased expression of it, especially of MnSOD [41], showed significantly-statistically higher activity in individuals from the melanoma group, compared to those from the control group (89.61 ± 14.89 vs. 71.72 ± 4.76; *p* < 0.05). The activity of the same enzyme was reduced, statistically significant, following the administration of rhodoxanthin in individuals with melanoma, untreated (75.97 ± 1.17 vs. 89.61 ± 14.89; *p* < 0.05). The low activity of antioxidant enzymes at the tumor level has been challenged by some researchers who have demonstrated an increased activity, particularly of MnSOD, in certain neoplasms such as mesothelioma, renal carcinoma, and ovarian cancer in humans.

Thus, an explanation of the increase in SOD activity in melanoma-bearing individuals, compared to those in the control group, respectively, the melanoma + rhodoxanthin group, may be the result of cellular response to intrinsic oxidative stress at the level of tumor cells. Similar to data from previous publications [41], the mean value of glutathione-peroxidase determined from the plasma of individuals with melanoma was lower compared to that of individuals from the control group. The mean value of GPx corresponding to individuals in the melanoma + rhodoxanthin group was higher than that of melanoma-bearing individuals, untreated, but this increase was not statistically significant (*p* > 0.05). Similarly, a higher value of GPx was determined from the plasma of individuals in the rhodoxanthin group, compared to the untreated control group, a value close to that of individuals in the melanoma + rhodoxanthin group (25.72 ± 5.07 vs. 25.34 ± 0.58).

Total antioxidant status represents the total antioxidant effect of all antioxidant defense systems, enzymatic and non-enzymatic, at the circulatory level. Thus, it can be considered that this parameter is closely related to the exogenous intake of antioxidants, through the diet, so a low value of this parameter can be interpreted as either a response to increased production of SRO, or a consequence of a deficient intake of antioxidants [42]. In our study, a slight decrease in TAS was observed in the melanoma group compared to the control group (17.30 ± 0.33 vs. 18.31 ± 1.68; *p* > 0.05), whereas the melanoma + rhodoxanthin group recorded a significant increase compared to both the melanoma group (30.33 ± 8.16 vs. 17.30 ± 0.33; *p* < 0.05) and the control group (30.33 ± 8.16 vs. 18.31 ± 1.68; *p* < 0.05). As a consequence, we can corroborate the previously suggested theory regarding the increase in TAS value following dietary supplementation with natural antioxidant chemicals.

**Figure 6:**
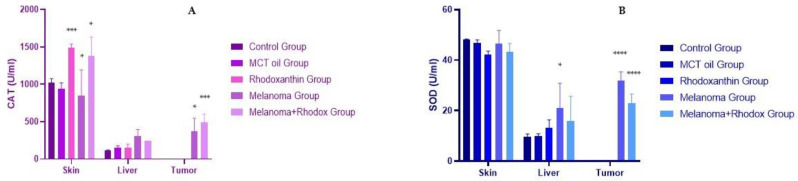
Enzymatic antioxidant activity of catalase (**A**) and superoxide-dismutase (**B**) in tissues. Values are expressed as mean ± standard deviation SD from three independent experiments. Statistical significance level * *p* < 0.05, *** *p* < 0.01 and **** *p* < 0.0001.

**Figure 7:**
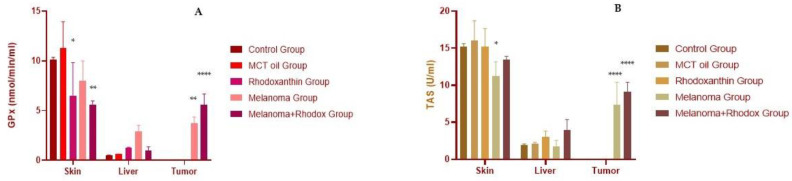
Enzymatic antioxidant activity of glutathione-peroxidase (**A**) and the total antioxidant status (**B**) in tissues. Values are expressed as mean ± standard deviation SD from three independent experiments. Statistical significance level * *p* < 0.05, ** *p* < 0.01, and **** *p* < 0.0001.

Table 2, Figure 6 and Figure 7 show the values of enzyme antioxidant activity, total antioxidant status, and total protein content of protein extracts obtained from the tissues harvested at the end of the study from the mice.

At the skin tissue level, we noticed an increase in catalase activity in the rhodoxanthin group compared to the value determined in the control group (*p* < 0.05). In addition, the melanoma + rhodoxanthin group had a higher catalase value than the control group and the melanoma group (1381.91 ± 250.06 vs. 1025.11 ± 50.14, *p* < 0.05; 1381.91 ± 250.06 vs. 846.23 ± 345.21, *p* < 0.05). In the same tissue, the SOD value did not differ significantly between groups, with a minor decrease in all other groups compared to the control group, with the melanoma + rhodoxanthin group having the lowest representative value.

Furthermore, the glutathione-peroxidase value decreased in the rhodoxanthin, melanoma, and melanoma + rhodoxanthin groups, with the melanoma + rhodoxanthin group having the lowest value (melanoma vs. control, *p* < 0.05). The TAS value showed the following variations: a drop in the melanoma group vs. the control group (*p* < 0.05), a minor decrease in the melanoma + rhodoxanthin group vs. the control group (*p* < 0.05), and an increase in the melanoma + rhodoxanthin group vs. the melanoma group (*p* > 0.05).

Significant changes in catalase were found in liver tissue, with an increase in activity in all groups compared to the control group (melanoma vs. control, *p* < 0.05). SOD activity at the liver level was also significantly increased in the rhodoxanthin, melanoma, and melanoma + rhodoxanthin groups. Similar information was gathered for the GPx value. The variance of the TAS values was slightly different, with the melanoma group registering the lowest value and the melanoma + rhodoxanthin group registering the greatest value (*p* > 0.05).

Catalase levels in tumor tissues were higher in patients treated with rhodoxanthin (492.22 ± 108.31 vs. 368.162 ± 178.48, *p* > 0.05). Similarly, GPx’s activity and TAS value were higher in the melanoma + rhodoxanthin group as compared to the melanoma group. The SOD value, on the other hand, was the only one that decreased following the administration of rhodoxanthin.

An explanation for the increased value of some of the antioxidant enzymes at the level of tumor cells or in the plasma of melanoma-bearing individuals can be based on the action of reactive oxygen species at the cellular level. Constantly, neoplastic cells adapt to the conditions, reprogramming their metabolism so that the energy requirements for growth and survival are met. One thing is certain thing is that the cells do not use a single adaptation strategy, instead they select among a variety of metabolic processes [43]. Thus, it is known that an increased accumulation of ROS leads to the activation of various signaling pathways and some mutations at the level of transcription factors, such as NF-Kb (cytoplasmic nuclear transcription factor). Nonetheless, even in neoplastic cells, an excessive accumulation of ROS will trigger severe cell damage, culminating in cell apoptosis. To counteract these effects, tumor cells have defense mechanisms by adapting to oxidative stress through an increased antioxidant response of the tumor, thus maintaining a level of ROS below the critical threshold, but still higher than in normal cells [44].

With respect to malignant melanoma, there is a plethora of research being conducted in both humans and animals. Contrary to popular belief and initial research findings, antioxidant enzyme levels in neoplastic cells are higher than those in non-neoplastic cells in tissues proximal to tumors. Some authors [45] reported an unexpected elevation in SOD levels in human melanoma samples when compared to melanoma-adjacent tissues and other melanocytic nevi samples. SOD was shown to be increased in murine melanoma tumors when compared to squamous cell carcinoma, basal cell carcinoma, and healthy skin tissue, all human samples, according to a study published in 2002 [46]. Another study surveyed C57BL/6J mice of various ages that were injected intraperitoneally with 10^6^ B16 melanoma cells, and discovered a substantial increase in the antioxidant enzymes SOD, GPx, and CAT evaluated from the liver tissue of these individuals when comparing these results with the ones obtained after analyzing the tissues from the healthy individuals. A comparable increase in enzymatic activity was seen in the brain and lungs of melanoma-bearing mice in the same investigation [47]. Another study [42] reported an increase in enzyme activity (CAT, SOD, GPx, and TAS) at the serum level in patients with malignant melanoma following a study that aimed to investigate the antioxidant status in dogs with various types of neoplasms, implying a tumor cell defense mechanism against the effects of ROS. Thus, the authors of the same study believe that increasing the level of antioxidant enzymes in patients with tumors may be due to a systemic increase in the production of ROS or to the tumor cells’ excessive production of O_2_^•−^, a situation in which the anions superoxide cross the erythrocyte membrane, stimulating the production of antioxidant enzymes.

### 3.4. Determination of 8OHdG and EGF Activity

The index of DNA oxidation, 8-hydroxy-2-deoxyguanosine (8OHdG), is considered an important factor to monitor in the case of neoplasms, and is also associated with determining the prognosis in the case of solid tumors [31]. It can be determined from plasma, tissues, and urine. Increased values of this marker are associated with permanently increased oxidative stress in tumor cells.

**Figure 8:**
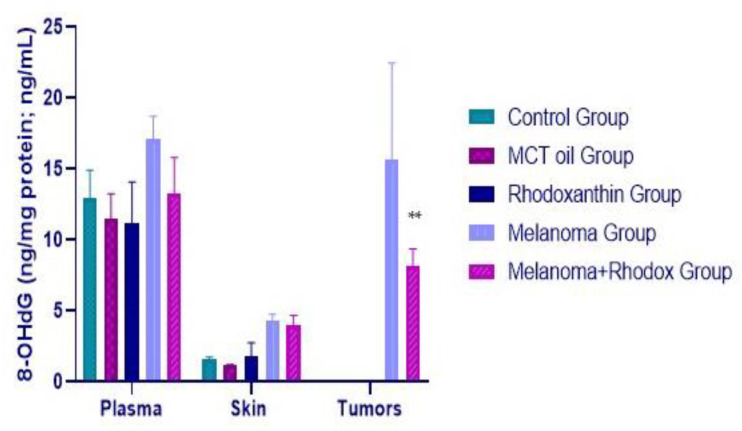
8-OHdG variation within the groups. Statistical significance level ** *p* < 0.01.

As can be seen in the data in Figure 8, the average value of this indicator, determined from the skin tissue level, in the case of the melanoma group is 2.84 times higher than the value corresponding to the control group (*p* < 0.05). In the case of the melanoma + rhodoxanthin group, a 16.32% decrease in the average value can be observed compared to that of the melanoma group (*p* > 0.05). Regarding the comparison of the results of the mean concentration of 8OHdG from the tumors, a marked difference was determined, although not statistically significant. Thus, the concentration of this marker is lower by 42.71% in the case of tumors collected from individuals treated with rhodoxanthin, compared to those collected from untreated individuals. This fact indicates a much lower concentration of DNA oxidation compounds at the level of tumor cells following treatment with rhodoxanthin, respectively, and a much lower accumulation of reactive oxygen species at this level.

The value of 8-OHdG determined from the plasma level varied as follows: an increase of 23.47% in the case of the melanoma group vs. control and a decrease of 13.54% in the case of the melanoma + rhodoxanthin group vs. melanoma (*p* < 0.05).

**Figure 9:**
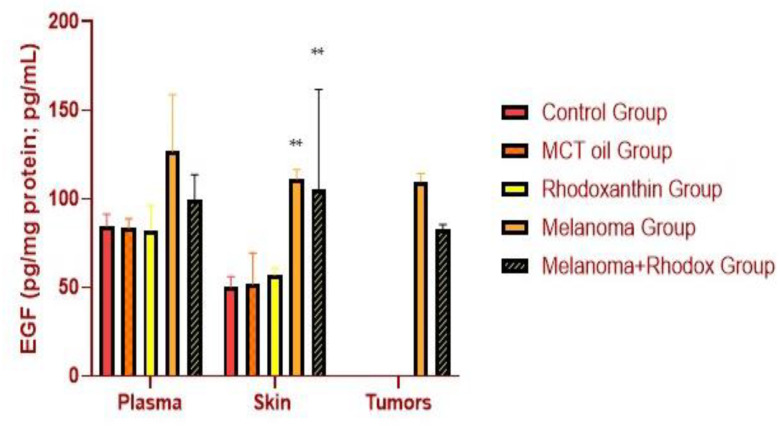
EGF variation within the groups. Statistical significance level ** *p* < 0.01.

Epidermal growth factor plays an important role in tumor initiation and progression through several mechanisms, the most important being the suppression of tumor cell apoptosis, thus promoting tumor growth. Figure 9 illustrates the effect of rhodoxanthin supplementation on the EGF value in the subjects investigated. This had a statistically significant value (*p* < 0.05) in the skin (where we observed a decrease of 18.6%) and at the tumor level (where we observed a decrease of 29.04% of the EGF activity as a result of rhodoxanthin intake). Furthermore, the determination of the EGF value in the plasma showed an important decrease, its value being lowered by 21.31% in the case of individuals with melanoma who were administered rhodoxanthin compared to the untreated tumor-bearing subjects (*p* < 0.05). This demonstrates the involvement of rhodoxanthin in slowing tumor growth, one of the mechanisms being the decrease of EGF levels.

### 3.5. Histological Analysis

**Figure 10:**
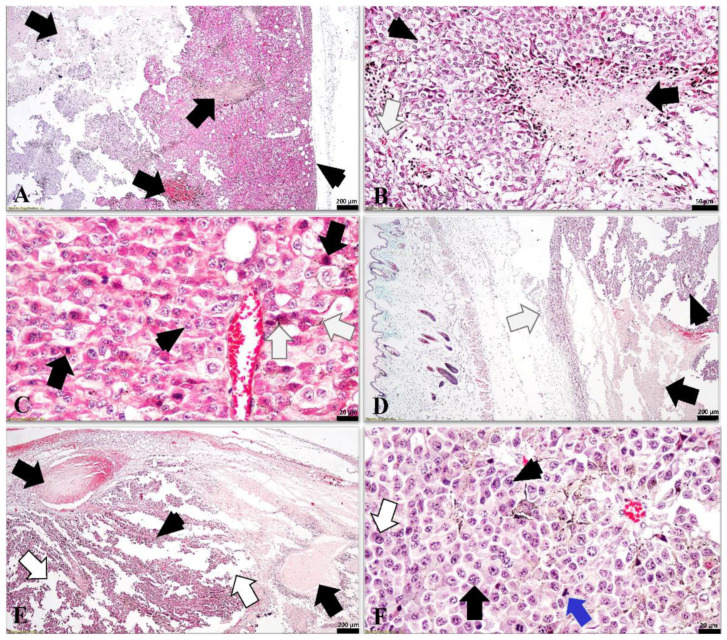
Cutaneous anaplastic melanoma in mice (Goldner’s trichrome stain): (**A**) (control group—M1): large tumoral mass with extended necrotic areas (arrows) and a discreete capsule (arrowhead); (**B**) (control group—M8): microscopic features of the melanoma suggesting cellular debris of the necrotized areas (black arrow), tumoral parenchyma (arrowhead) that includes a discrete sustaining tissue (white arrow); (**C**) (control group—M3): highly cellularised melanoma, with tumoral melanocytes displaying anisocytosis, large nuclei and nucleoli (arrowhead), numerous mitotic figures (black arrows), and discreete melanic granules in the cytoplasm of some tumoral cells (white arrows); (**D**) (group melanoma + rhodoxanthin—MR1): the location of the tumoral mass in the hypodermis (arrowhead), which presents large necrotic areas (black arrow) and an ill-defined infiltrated capsule (white arrow); (**E**) (group melanoma + rhodoxanthin—MR1): thrombi in the tumoral mass (arrows), a discrete sustaining tissue identified in between the parenchyma cells (arrowhead) and a low intercellular adhesion suggested by large intercellular spaces (white arrows) suggesting rapid growth and high malignancy; (**F**) (group melanoma + rhodoxanthin—MR8): poorly differentianted melanoma suggested by high cellular pleiomorphism, large nuclei (black arrow) including binucleated melanocytes (white arrow), in many circumstances with macronucleoli (arrowhead), high mitotic index that includes atypic mitoses (blue arrow).

A poorly differentiated malignant lesion was identified in the analyzed samples, located in the dermis and hypodermis (Figure 10). Tumor cells displayed a high polymorph appearance (high cellular polymorphism), including cells with a polygonal appearance and spindle or dendritic-shaped appearance. The neoplastic cells showed large round to ovoid nuclei (6–9 times the size of red blood cells), in the vast majority of cases, the nuclei presented an irregular outline, including invaginations of the nuclear envelope. The nucleoli ranged from 1 to 3 in number, and most of them were uneven and large, some tumoral cells displaying bizarre appearances. The cytoplasm of some tumoral cells was poorly charged with a brown to dark-brown granular melanin, whereas the cytoplasm had an acidophilic or foamy appearance. Intratumoral mitoses were frequently detected, some of them with an atypical appearance. However, scattered apoptotic cells were identified in the mass of the tumor. Due to the rapid pace of tumor development, the sustaining tissue is poorly represented by delicate collagen strands that include blood vessels, and, as a consequence, the intercellular adhesion of the tumoral parenchyma cells was poor. Most of the assessed melanomas in both groups showed several necrotic microfoci or large necrotic areas, suggesting once again a rapid growth ratio and an increased degree of anaplasia. All analyzed tumors presented a discreet capsule, often infiltrated mostly by lymphocytes, histiocytes, and/or tumoral cells. The histopathological diagnosis was cutaneous anaplastic malignant melanoma.

## 4. Conclusions

Supplementing the C57BL/6J mice diet with rhodoxanthin did not result in the appearance of any specific symptoms of intoxication, the weight of the individuals included in the study remained constant during the 21 days. Following the subcutaneous injection of 10^6^ B16F10 cells, both the mice in the melanoma group and those in the melanoma + rhodoxanthin group developed tumors. The administration of rhodoxanthin visibly slowed down this development determining variations in antioxidant enzymes, both at the plasma level and at the tissue level. The value of EGF activity determined from plasma and tissues was major in the case of the melanoma group, with a significant decrease in the case of individuals treated with rhodoxanthin, and the variation of 8-OhdG concentration was similar to that of EGF. We can conclude by emphasizing the significant effect of reducing tumor growth by supplementing the diet with rhodoxanthin extracted from the aril of *Taxus baccata*, in the case of murine melanoma induced subcutaneously in CD57BL/6J mice. Nevertheless, further investigations on other cell lines are still necessary to confirm the antitumor potential of the extract.

## Figures and Tables

**Table 1:** Blood count evaluation.

Parameters	Control Group	MCT Oil Group	Rhodoxanthin Group	Melanoma Group	Melanoma + Rodhox Group
WBC (10^9^/L)	3.603 ± 0.14	3.977 ± 0.03	5.056 ± 0.04	5.777 ± 0.12	3.626 ± 0.05
LYM (10^9^/L)	3.253 ± 0.03	3.466 ± 0.21	4.166 ± 0.03	4.036 ± 0.07	2.850 ± 0.05
NEU (10^9^/L)	0.253 ± 0.04	0.476 ± 0.02	0.853 ± 0.04	1.473 ± 0.03	0.793 ± 0.04
RBC (10^12^/L)	8.593 ± 0.03	10.096 ± 0.02	10.826 ± 0.06	4.332 ± 0.05	8.250 ± 0.05
HGB (g/dL)	11.210 ± 0.04	13.876 ± 0.08	14.486 ± 0.09	10.943 ± 0.05	11.260 ± 0.03
HCT (%)	38.926 ± 0.41	41.713 ± 0.28	44.210	33.916 ± 0.13	35.010 ± 0.11
PLT (10^9^/L)	252.12 ± 1.42	231.943 ± 0.41	184.5 ± 0.51	91.333 ± 1.52	230.643 ± 0.66

(WBC—white blood cells; LYM—lymphocytes; NEU—neutrophils; RBC—red blood cells; HGB—hemoglobin; HCT—hematocrit; PLT—platelets).

**Table 2:** Total protein evaluation of tissues.

Tissue/Parameters	Total Proteins (mg/mL)
Skin	
Control group	1.39 ± 0.28
MCT oil group	1.53 ± 0.39
Rhodoxanthin group	1.08 ± 0.03
Melanoma group	2.05 ± 0.89
Melanoma + Rhodoxanthin group	1.102 ± 0.13
Liver	
Control group	14.30 ± 0.28
MCT oil group	10.56 ± 1.89
Rhodoxanthin group	8.71 ± 1.201
Melanoma group	5.94 ± 1.22
Melanoma + Rhodoxanthin group	5.50 ± 2.15
Tumor	
Melanoma group	3.18 ± 0.62
Melanoma + Rhodoxanthin group	3.19 ± 0.44

## Data Availability

The data presented in the study are available in the article.
